# Pennsylvania State Board of Veterinary Medical Examiners

**Published:** 1896-05

**Authors:** 


### PENNSYLVANIA STATE BOARD OF VETERINARY MEDICAL EXAMINERS.

THE Pennsylvania State Board of Veterinary Medical Examiners held their first examination at Philadelphia on April 20th and 21st. By courtesy of the Mayor of the city, the examinations were held in the civil-service examining-rooms in the City Hall, and afforded every facility for the completion of the work as well as the comfort and convenience of the applicants. The examination commenced at 10 A.M. each day and lasted until 6 P.M., with an hour recess at noon. The entire board was present.

Some fourteen applicants presented themselves, representing the Ontario Veterinary College, New York Veterinary College, and National Veterinary College. Some thirteen subjects for examination, designated by the law, were grouped to make eleven and were all written; fifteen questions were submitted on each subject, ten to be answered.

Of the fourteen applicants three were successful and eleven failed. The names of those passing and who received the license of the board were as follows: Dr. Elmer Seitz, Seitzland, Pa., graduate of the National Veterinary College; Dr. W. R. Jobson, Franklin, Pa., graduate of the National Veterinary College; Dr. Wm. S. Longacre, Mantz, Schuylkill County, Pa., graduate of the Ontario Veterinary College.

The Board after thorough consideration of the matter decided to leave it optional with the applicants who failed as to whether they should elect to take over again the whole examination, or only those subjects on which they had failed. In the event of choosing the latter the markings on the subjects passed to count in computing the general average (65 per cent.) to entitle to license.

The Board authorized the immediate prosecution of three violators of the Act, and decided upon the most vigorous action in dealing with all violations of the several provisions of the Act. Every prothonotary in each county of the State has been personally notified to register none without a license of the Board, bearing the State seal; and in every county of the State a qualified veterinarian will be designated to look after any violators or violations of the provisions of the Act. An arrest has been made in York County, and the violator is now under bail, and the most vigorous prosecution will be pursued in sustaining the Act, in order that the value of the provisions of this law shall be fully tested.

